# Emergence of Two New Lineages of the Cosmopolitan Dengue Virus Serotype 2 in Yunnan, China, 2024

**DOI:** 10.1093/infdis/jiaf422

**Published:** 2025-08-11

**Authors:** Man Li, Fen Zeng, Wei Chang, Mengyuan Zheng, Xiaojuan Chen, Ziying Wu, Li Liu, Xueshan Xia, Yue Feng

**Affiliations:** Faculty of Life Science and Technology, Kunming University of Science and Technology, Kunming, Yunnan, China; Yunnan Provincial Key Laboratory of Public Health and Biosafety, Kunming Medical University, Kunming, Yunnan, China; Department of Infectious Diseases, Xishuangbanna Dai Autonomous Prefecture People's Hospital, Xishuangbanna, Yunnan, China; Faculty of Life Science and Technology, Kunming University of Science and Technology, Kunming, Yunnan, China; Faculty of Life Science and Technology, Kunming University of Science and Technology, Kunming, Yunnan, China; Department of Infectious Diseases, Xishuangbanna Dai Autonomous Prefecture People's Hospital, Xishuangbanna, Yunnan, China; Department of Infectious Diseases, Xishuangbanna Dai Autonomous Prefecture People's Hospital, Xishuangbanna, Yunnan, China; Faculty of Life Science and Technology, Kunming University of Science and Technology, Kunming, Yunnan, China; Yunnan Provincial Key Laboratory of Public Health and Biosafety, Kunming Medical University, Kunming, Yunnan, China; Faculty of Life Science and Technology, Kunming University of Science and Technology, Kunming, Yunnan, China; Yunnan Provincial Key Laboratory of Public Health and Biosafety, Kunming Medical University, Kunming, Yunnan, China

**Keywords:** Dengue virus, serotypes, genetic diversity, cosmopolitan, new lineage

## Abstract

**Background:**

Dengue fever has become a significant epidemic in South and Southeast Asia. Recently, Yunnan Province in southwestern China, which borders these regions, has also experienced a significant outbreak. This study aims to investigate the genetic diversity of the dengue virus (DENV) and identify potential sources of transmission in Xishuangbanna, Yunnan Province in 2024.

**Methods:**

In 2024, we investigated 321 suspected cases of dengue fever in Yunnan Province. We screened dengue fever patients using a combination of DENV NS1 antigen and Pan-qPCR methods, and performed serotype typing using specific qPCR. We also performed phylogenetic and molecular clock analyses to understand the genotypes and transmission sources.

**Results:**

DENV infection was detected in 101 of 321 specimens. Serotype and genotype analysis were performed on 88 of the 98 positive samples. The results showed that 81 cases belonged to the cosmopolitan genotype and cosmopolitan_asian-pacific subtype of DENV serotype 2 (DENV-2), while only 1 case was classified as genotype I and subtype 1L of DENV serotype 1 (DENV-1). Notably, samples within the cosmopolitan_asian-pacific subtype were primarily divided into 2 distinct lineages. Further analysis of whole-genome sequences, focusing on phylogenetic history and spatiotemporal dynamics, indicated that lineage-1 likely originated in Thailand, while lineage-2 may have originated in Cambodia. In addition, we analyzed all available DENV-2 sequences from Yunnan in the GenBank database and found that DENV-2 genotypes in the Xishuangbanna region were more diverse and complex.

**Conclusions:**

This study identifies 2 new lineages of cosmopolitan DENV-2 in Yunnan, originating from Thailand and Cambodia, respectively.

## Teaser

This study reports on 2 new cosmopolitan lineages of DENV-2 in Yunnan, China, in 2024, tracing them back to their origins in Thailand and Cambodia. It is essential to strengthen cross-border dengue fever prevention and control measures in Yunnan.

Dengue is the most prevalent mosquito-borne viral infection and usually presents with high fever, headache, muscle pain, joint pain, and vomiting [1, 2]. It is endemic in over 120 countries across tropical and subtropical regions of Southeast Asia, Africa, the Western Pacific, and the Americas [3]. The global burden of dengue has continued to increase over the past decade, characterized by significant outbreaks in endemic areas and a growing number of cases among travelers [4, 5]. Currently, there are no approved antiviral drugs or widely available vaccines for dengue [6]. Prevention efforts focus on reducing disease incidence.

Yunnan Province in China experiences tropical and subtropical climates and shares a 4060 km border with neighboring countries, Vietnam, Laos, and Myanmar [7]. These countries have endemic dengue fever, which presents a potential risk for Yunnan Province. Since 2013, a significant dengue outbreak has occurred in the Xishuangbanna and Dehong Prefectures of Yunnan, which border Laos and Myanmar [8]. Climate change, population growth, human mobility, and urbanization are expected to increase the burden of dengue fever [1].

Xishuangbanna, a tourist city located in Yunnan province near the borders of Myanmar and Laos, attracts numerous visitors yearly due to its unique climate and geographical features [7]. However, this same climate is also ideal for the growth and reproduction of *Aedes mosquitoes*, making Xishuangbanna one of the primary regions where dengue fever is prevalent. From 2013 to 2019, there were new cases and regional outbreaks of dengue virus (DENV) nearly every year in Xishuangbanna [9]. Specifically, confirmed cases were reported as follows: 1262 cases in 2013, 1131 cases in 2015, 1184 cases in 2017, and over 3900 cases in 2019 [7–10]. In this study, we investigated the molecular epidemiological characteristics of dengue fever in the Xishuangbanna region in 2024. We also systematically analyzed the epidemiological and evolutionary attributes of dengue type 2 in Yunnan. This research provides a basis for effectively preventing and controlling dengue type 2 virus in Yunnan.

## METHODS

### Study Participants

**Figure 1.**
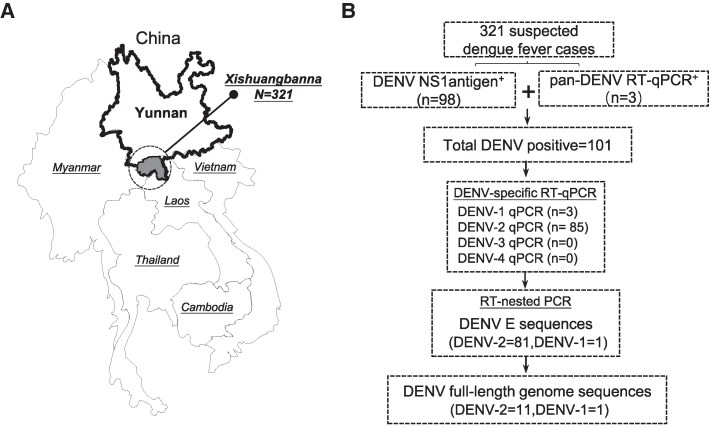
Sampling sites and study design for this research. (*A*) The location of the Xishuangbanna region in Yunnan Province, China. (*B*) The study design and subsequent analysis.

From June to October 2024, a total of 321 serum samples from patients with suspected acute dengue infection were tested at the Xishuangbanna Dai Autonomous Prefecture People's Hospital in Yunnan, China (Figure 1*A*). All specimens were collected from individuals presenting with symptoms consistent with acute dengue infection within the first 5 days of illness. The samples were then transported to our laboratory facility, where the serum was separated and stored at −80°C.

### DENV NS1 Antigen Testing, RNA Extraction, DENV Gene Amplification, and Sequencing

The DENV antigen rapid test was performed using DENV NS1 antigen test strips (Wantai, Beijing, China). RNA was extracted from 200 µL of serum samples using the MiniBEST Viral RNA/DNA Extraction Kit (Tiangen, Beijing, China) according to the manual's instructions. Figure 1*B* illustrates the procedure for detecting and genotyping DENV. First, 321 samples were screened using DENV NS1 antigen test strips, yielding 98 positive results. Next, the remaining 223 NS1-negative samples were tested using a pan-reverse transcription (RT)-DENV quantitative PCR (qPCR) assay [11], identifying 3 additional DENV RNA-positive cases. Serotyping was then performed on the 101 DENV-positive samples using 4 serotype-specific RT-qPCR assays [12]. Based on the serotyping results, RT-nested PCR amplification and sequencing of the DENV E1 and full-length genome sequences were carried out [13, 14]. Amplified PCR products were detected by electrophoresis on a 1.0% agarose gel under ultraviolet illumination and purified using a DNA purification kit (Tiangen, Beijing, China). The products were sequenced by TSINGKE Biological Technology Co. on an ABI 3730XL automated DNA Sequencer. This study identified 85 cases of DENV-2 and 1 case of DENV-1 in the serum of 101 patients diagnosed with dengue fever. A total of 81 DENV-2 E1 sequences, 1 DENV-1 E1 sequence, and 12 complete DENV genome sequences were obtained.

### Phylogenetic Analysis

NCBI BLASTn was used to identify the strain most closely related to our samples, and representative sequences were retrieved for phylogenetic analysis. Multiple sequence alignments were performed using the online version of MAFFT (v7.0) with the default parameters. A maximum likelihood phylogeny was constructed from this dataset. The phylogenetic model and tree inference were performed simultaneously using IQ-TREE v2.0.3, with 1000 ultrafast bootstrap replicates. The GTR + I + G model was determined to be the best-fitting nucleotide substitution model for the dataset.

### Bayesian Markov Chain Monte Carlo Evolutionary Analyses

Bayesian coalescent analysis using the Markov chain Monte Carlo (MCMC) sampling method was performed through BEAST version 1.10.4 under the uncorrelated log-normal relaxed clock model with the GTR + I + G nucleotide substitution model, a Bayesian Skyline coalescent tree prior, and a relaxed uncorrelated log-normal molecular clock model. Each MCMC analysis was executed for 30 million states, sampled at every 30 000 states. The model with an effective sampling size > 200 was selected. Posterior probability densities were determined in Tracer v1.5 (http://tree.bio.ed.ac.uk/software/tracer/), and 10% of each chain will be discarded as burn-in. The maximum clade credibility (MCC) summary tree was generated using TreeAnnotator v1.10.4 from the BEASTv1.10.4 software package, with the initial 10% of trees discarded as burn-in. The resulting MCC summary tree was rendered in FigTree software for subsequent analysis.

### Statistical Analysis

Continuous variables were described as mean ± standard deviation, while categorical variables were presented as percentages with 95% confidence intervals (CIs). The Kruskal–Wallis test was used to compare the median age of dengue case patients, both negative and confirmed, and the Pearson χ^2^ test was used when appropriate to compare percentages between categories. A *P* value <.05 was considered statistically significant. All data analyses were performed using SPSS software version 22.0.

### GenBank Accession Numbers

The nucleotide sequences reported in this study have been submitted to GenBank under accession numbers PV012231-PV012241 for the 11 complete DENV-2 genomes, PV000786 for the single complete DENV-1 genome, and PV093956-PV094036 for all 81 partial E1 sequences.

### Ethics Statement

This study was performed in line with the principles of the Declaration of Helsinki for Human Research of 1974. All participants provided written informed consent for sample collection and subsequent analyses, and the study was approved by the Medical Ethics Committee of Kunming University of Science and Technology No. KUST-MEC-097.

## RESULTS

### Epidemiological Characteristics

In this study, the overall prevalence of DENV infection was detected in 101 of the 321 samples (31.5%, 95% CI 26.4%–36.5%). The mean age of all patients was 45.5 ± 13.8 years, with the majority (50.0%) of participants falling within the >45-year age group, followed by the 30–45-year (22.4%) and 18–29-year (20.4%) age groups. The DENV positivity rate significantly varied according to age group (*P* = .013). To gain a better understanding of the age distribution of dengue virus infections [15], we counted the number of cases and their serotype distribution in each age group every month (Supplementary Figure 1). A higher prevalence of dengue infection was observed among individuals of the Han nationality compared with the Dai nationality, with a ratio of 2:1 for confirmed cases (*P* = .002). Among the confirmed dengue cases, the most prevalent symptoms reported were headaches (87.1%), followed by myalgia (55.4%), arthralgia (31.7%), and vomiting (31.7%) (Table 1). According to the diagnostic criteria outlined in the Chinese Dengue Fever Diagnosis and Treatment Guidelines (2024), the cases were classified as mild, and no differences in clinical severity were observed among the patients in this study.

**Table 1.** Epidemiologic and Clinical Characteristics of Suspected and Confirmed Dengue Fever Case-patients in Xishuangbanna, China, 2024

Variables	Total (*n* = 321)	DENV Positive (*n* = 101)	DENV Negative (*n* = 220)	*P* Value
Sex, *n* (%)	…	…	…	.546
Female	143 (45)	42 (42)	101 (46)	
Male	178 (55)	59 (58)	119 (54)	
Age, *n* (%)	…	…	…	**.013**
<18	14 (4)	7 (7)	7 (3)	
18–29	79 (25)	20 (20)	59 (27)	
30–45	89 (28)	22 (22)	67 (30)	
>45	132 (41)	52 (51)	80 (36)	
Unknown	7 (2)	0 (0)	7 (3)	
Minorities, *n* (%)	…	…	…	**.002**
Han	173 (54)	67 (66)	106 (48)	
Dai	128 (40)	33 (33)	95 (43)	
Unknown	20 (6)	1 (1)	19 (9)	
Clinical symptoms	…	…	…	
Fever, *n* (%)	…	…	…	.895
Fever (≤39 °C)	293 (91)	93 (92)	200 (91)	
Fever (>39 °C)	28 (9)	8 (8)	20 (9)	
Headache, *n* (%)	…	…	…	.402
Yes	288 (90)	88 (87)	200 (91)	
No	33 (10)	13 (13)	20 (9)	
Myalgia, *n* (%)	…	…	…	.628
Yes	170 (53)	56 (55)	114 (52)	
No	151 (47)	45 (45)	106 (48)	
Arthralgia, *n* (%)	…	…	…	.49
Yes	112 (35)	32 (32)	80 (36)	
No	209 (65)	69 (68)	140 (64)	
Asthenia, *n* (%)	…	…	…	.499
Yes	22 (7)	5 (5)	17 (8)	
No	299 (93)	96 (95)	203 (92)	
Retroorbital pain, *n* (%)	…	…	…	.499
Yes	22 (7)	5 (5)	17 (8)	
No	299 (93)	96 (95)	203 (92)	
Rash, *n* (%)	…	…	…	.199
Yes	19 (6)	9 (9)	10 (5)	
No	302 (94)	92 (91)	210 (95)	
Vomiting, *n* (%)	…	…	…	.49
Yes	112 (35)	32 (32)	80 (36)	
No	209 (65)	69 (68)	140 (64)	
Time of Symptom Onset, d	…	…	…	.658
1	83 (26)	27 (27)	56 (25)	
2	80 (25)	21 (21)	59 (27)	
3	92 (29)	28 (28)	64 (29)	
4	49 (15)	19 (19)	30 (14)	
5	17 (5)	6 (6)	11 (5)	

Bold numbers indicate significant differences.

### Genotype Identification of DENV Isolates

**Figure 2.**
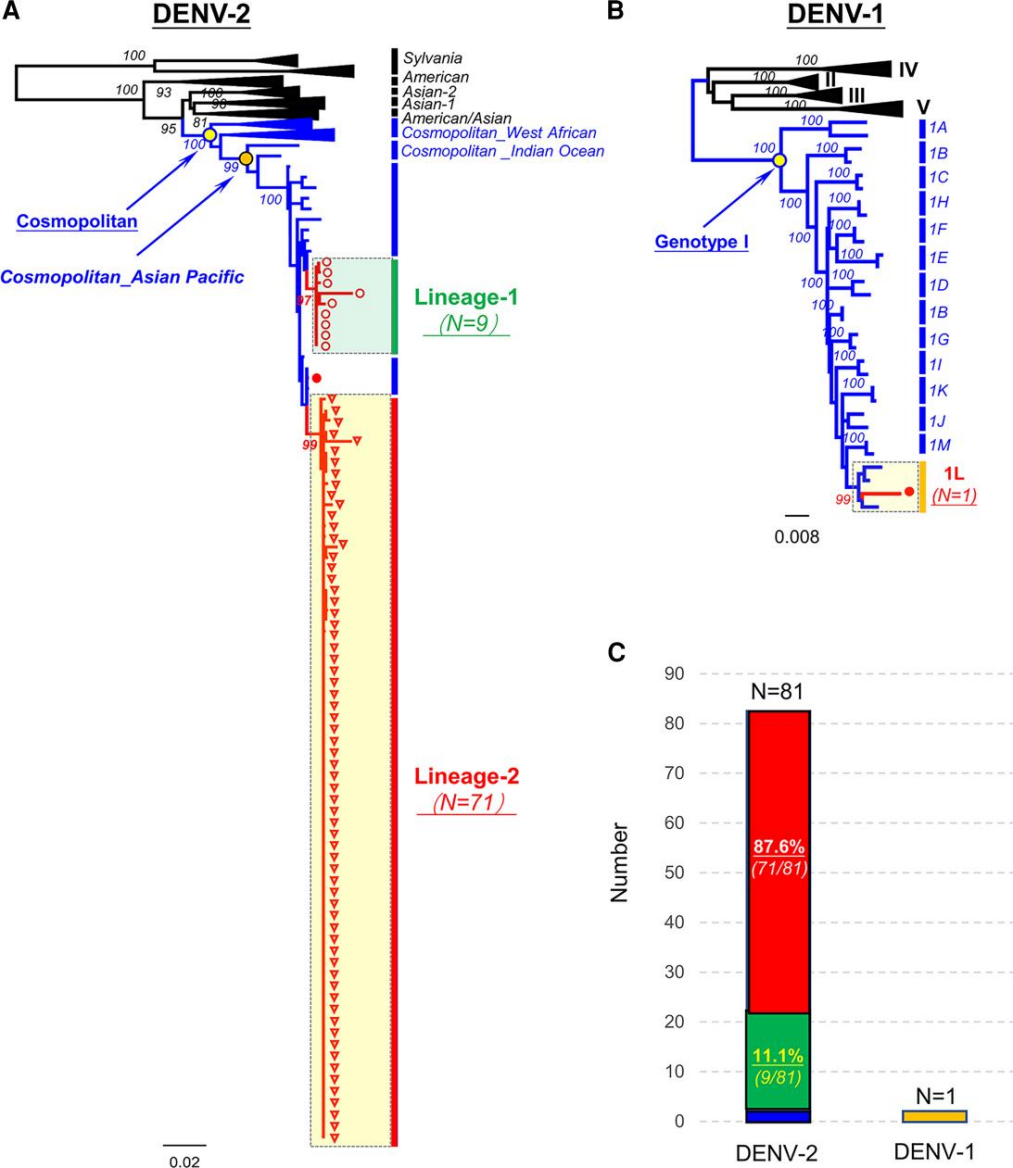
Phylogenetic analysis of DENV-1 and DENV-2 genotypes and their percentage distributions. (*A*) The maximum likelihood phylogenetic tree was constructed using reference sequences representing 6 DENV-2 genotypes: Sylvanian, American, Asian-1, Asian-2, American/Asian, and Cosmopolitan. The sequences analyzed in this study are highlighted in red. The stability of nodes in the tree was assessed using bootstrap analysis with 1000 replications. (*B*) A maximum likelihood phylogenetic tree was also constructed for DENV-1 using reference sequences representing 5 genotypes (I-V) and 1 subtype (1A-1 M). (*C*) The percentage distribution of each genotype within the DENV-1 and DENV-2 groups is shown.

We analyzed the phylogenetic tree based on the E region to further investigate the DENV genotypes. Our results showed that 1 sample of DENV-1 were classified under genotype I and subtype 1L (Figure 2*A*). In addition, of the 81 DENV-2 samples we examined, we found that they were distributed between the cosmopolitan genotype and the cosmopolitan_asian-pacific (C_asian-pacific) subtype (Figure 2*B*). Notably, the samples within the C_asian-pacific subtype were primarily divided into 2 distinct lineages: 9 samples (11.1%) were classified as lineage 1, whereas 71 samples (87.6%) were classified as lineage 2 (Figure 2*C*).

### Phylogeography of DENV2 Cosmopolitan Genotype

**Figure 3.**
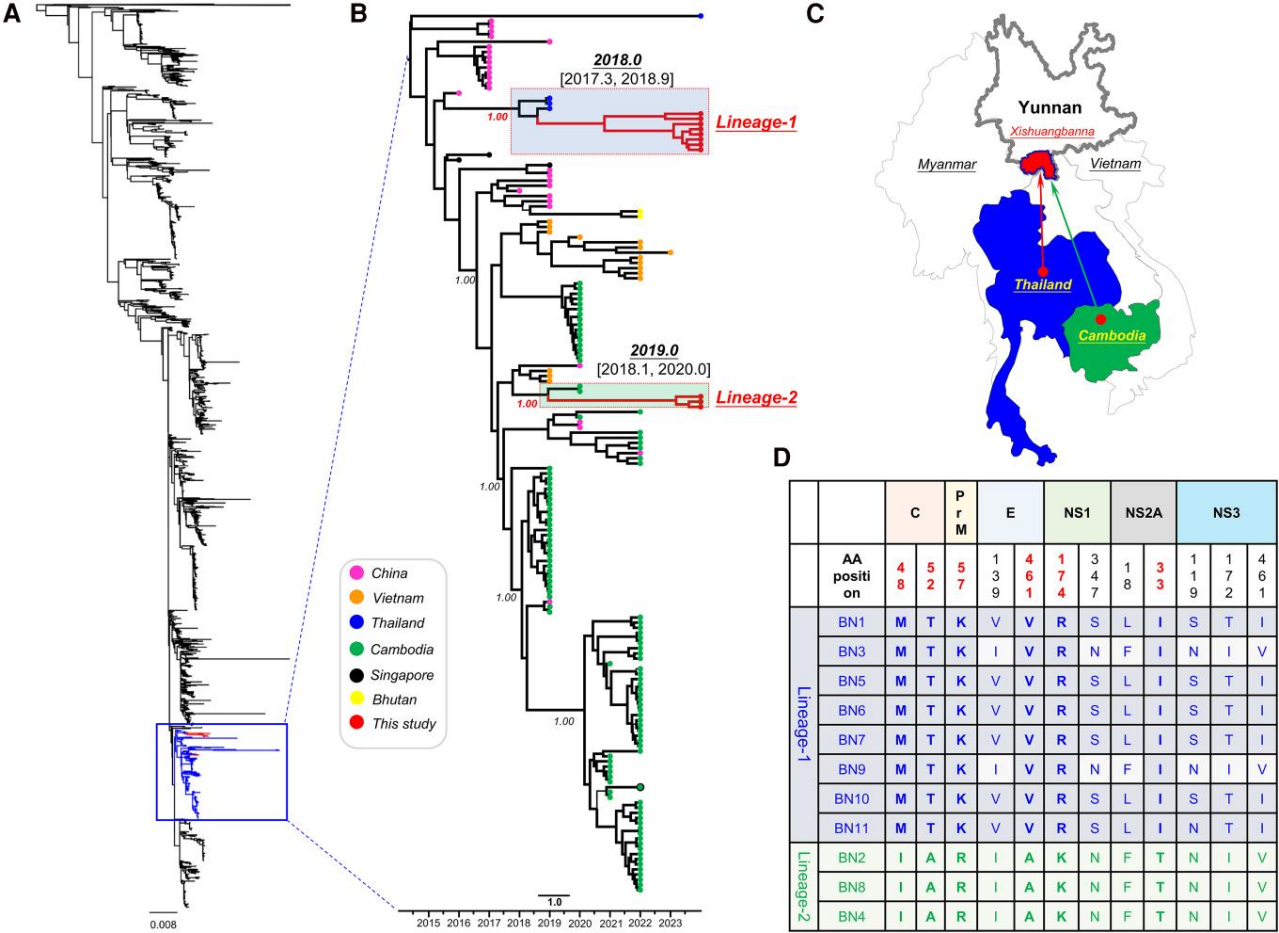
Spatio-temporal dynamics of 2 new lineages of cosmopolitan DENV-2 identified in 2024 in Yunnan, China, and a comparison of their amino acid variability. (*A*) The maximum likelihood tree includes the 2 new lineages of DENV-2 sequences (*n* = 11) generated in this study, along with a total of 2640 full-length genome reference strains belonging to the cosmopolitan genotype of DENV-2 from GenBank. (*B*) The inset tree on the right shows a maximum clade credibility (MCC) tree that estimates divergence times using a smaller dataset (*n* = 173) that includes all the new genomes from Yunnan. (*C*) This study identifies the potential countries of origin for the 2 new lineages found in the Xishuangbanna region of Yunnan Province. (*D*) A differential comparison of the whole-genome amino acid sequences of the 2 new DENV lineages is provided.

To understand the phylogenetic history of new lineages of the cosmopolitan DENV-2, we successfully obtained 11 whole-genome sequences from 8 samples belonging to lineage 1 and 3 samples from lineage 2. These 11 new sequences were combined with all available full-length genomes of DENV-2, genotype cosmopolitan, from GenBank, resulting in a dataset of 2640 sequences. Our analysis revealed that these 11 sequences were classified as the C_asian-pacific subtype and clustered within the extensive Southeast Asian group. Furthermore, the 11 sequences from this study formed 2 distinct monophyletic clades, suggesting that 2 independent introduction events likely occurred in Xishuangbanna, Yunnan, China (Figure 3*A*).

To further investigate the spatial-temporal dynamics of the 2 new lineages, a detailed analysis was conducted using a smaller dataset (*n* = 173) derived from the major Southeast Asian cluster (Figure 3*A*). Within the Xishuangbanna sequences, 2 distinct lineages were identified. Lineage-1, which dates back to late 2018.0 [95% HPD: 2017.3 to 2018.9], consists of 8 newly identified Xishuangbanna strains from lineage 1 that cluster with 3 strains isolated in Thailand. This observation suggests the possibility of virus introduction from Thailand to Xishuangbanna. Lineage-2, dating back to 2019.0 [2018.1, 2020.0], is comprised of 3 newly identified Xishuangbanna strains from lineage 2 that cluster with 2 strains isolated in Cambodia, suggesting a probable introduction from Cambodia to Xishuangbanna (Figure 3*B*). The 2 new lineage DENV-2 samples identified as endemic in Xishuangbanna, Yunnan, in 2024 are believed to have originated from 2 different Southeast Asian countries (Figure 3*C*).

To characterize the sequence variation features of the 2 newly discovered DENV lineages, the mean genetic distance within the amino acid sequences of the entire DENV genome was calculated. The results showed that the genetic distance of the sequence genes in lineage-1 was 0.56% ± 0.00076, greater than the 0.16% ± 0.00057 observed in lineage-2. Additionally, we compared the amino acid sequences encoded by DENV-2 for the 2 identified lineages using the BN1 sequence from lineage-1 (GenBank accession number PV012231) as a reference. We detected mutations at 12 amino acid sites in the C, PrM, E, NS1, NS2A, and NS3 genes (Figure 3*D*). Specifically, we identified 6 lineage-specific mutations in the C, PrM, E, NS1, and NS2A genes. For the lineage-1 strain, these mutations included 48M and 52T in C, 57K in PrM, 461V in E, 174R in NS1, and 33I in NS2A. In contrast, lineage 2 showed mutations at the same sites with the following changes: 48I and 52A in C, 57R in PrM, 461A in E, 174K in NS1, and 33T in NS2A.

### Diversity and Distribution of Dengue Serotype 2 in Yunnan

**Figure 4.**
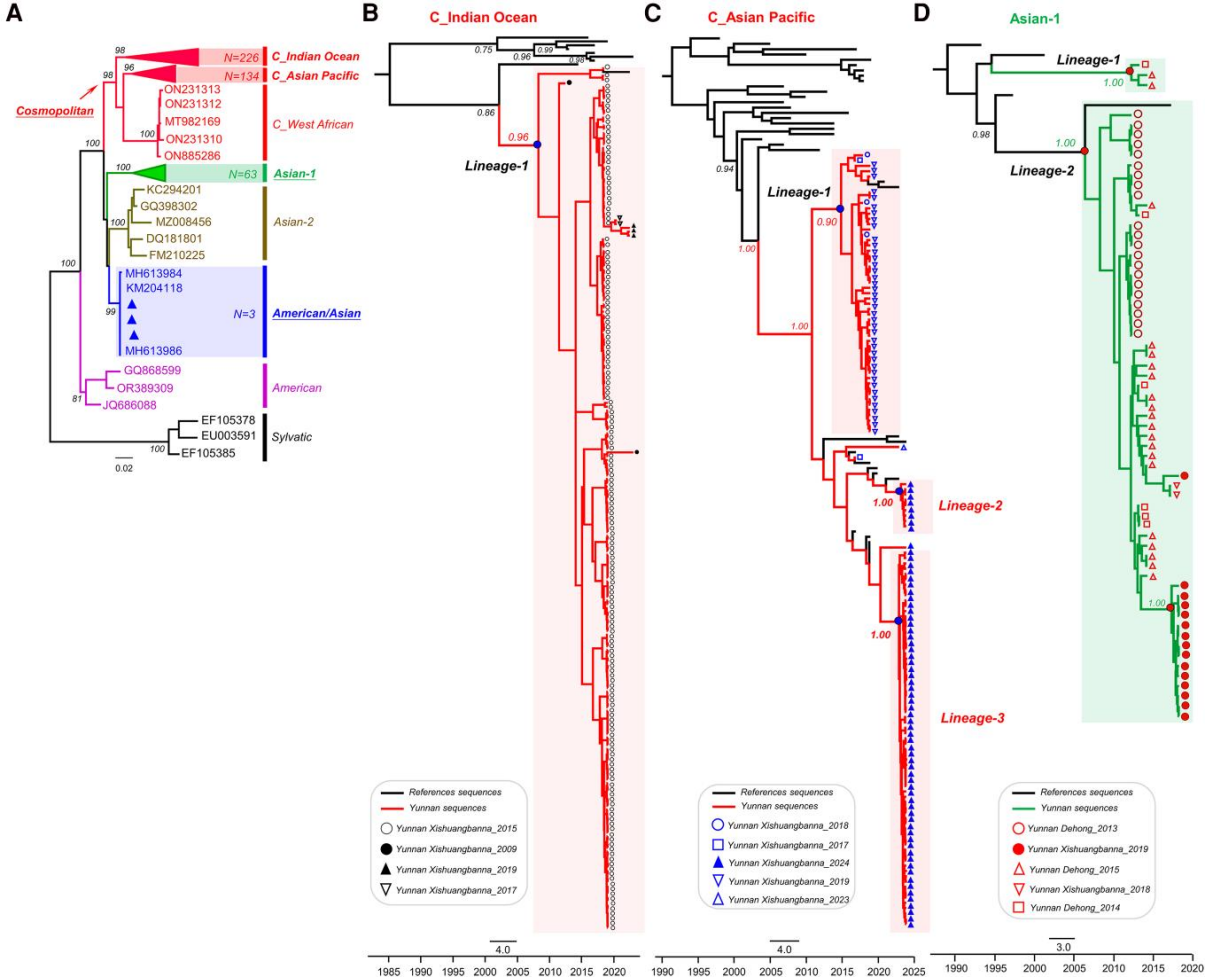
Phylogenetic tree analysis of different DENV-2 genotypes in Yunnan, China. (*A*) The 81 DENV-2 E-gene sequences obtained in this study were combined with a maximum likelihood phylogenetic tree constructed using all available 345 DENV-2 E-gene sequences from the Yunnan region in GenBank. (*B*) Maximum clade credibility (MCC) tree of the C_Indian Ocean lineage. (*C*) MCC tree of the C_Asian Pacific lineage. (*D*) MCC tree of the Asian-1 genotype. Sequences collected from different periods and sampling locations are indicated by unique identifiers in the lower left corner of the evolutionary tree.

To systematically characterize the diversity of DENV-2 in Yunnan, a total of 345 sequences of Yunnan DENV-2 E gene sequences from GenBank, as of 30 December 2024, were collected. These were then combined with 81 additional sequences from this study, resulting in a comprehensive dataset of 426 sequences. The basic information for all sequences is detailed in Supplementary Table 1. Further, Maximum Likelihood tree analysis revealed that the DENV-2 in Yunnan is predominantly represented by the Cosmopolitan strain (84.5%, 360), followed by the Asian-1 strain (15.0%, 63) and the American/Asian strain (0.5%, 3). Within the Cosmopolitan category (*n* = 360), 2 main subtypes were identified: the cosmopolitan_Indian Ocean (C_Indian Ocean) subtype, comprising 62.8% (*n* = 226), and the C_asian-pacific subtype, accounting for 37.2% (*n* = 134) (Figure 4*A*).

The phylogeny of DENV-2 strains inferred from a maximum credible clade tree shows 1 major lineage in the C_Indian Ocean subtype, 3 lineages in the C_asian-pacific subtype, and 2 lineages in the Asian-1 genotype (Figure 4*B*-*D*). The lineage in the C_Indian Ocean subtype mainly includes sequences from the Xishuangbanna region collected in 2015 (Figure 4*B*). Lineage 1 in the C_Asian Pacific subtype consists of sequences from Xishuangbanna collected in 2019, while lineages 2 and 3 include 2 newly discovered lineages from the same region identified in this study in 2024 (Figure 4*C*). Lineage 1 in the Asian-1 genotype contains only 3 sequences from the Dehong region collected in 2014 and 2015. In contrast, the sequences in lineage 2 have a more complex origin, including notable sequences from Dehong in 2013 and 2015, as well as sequences from Xishuangbanna collected in 2019 (Figure 4*D*).

**Figure 5.**
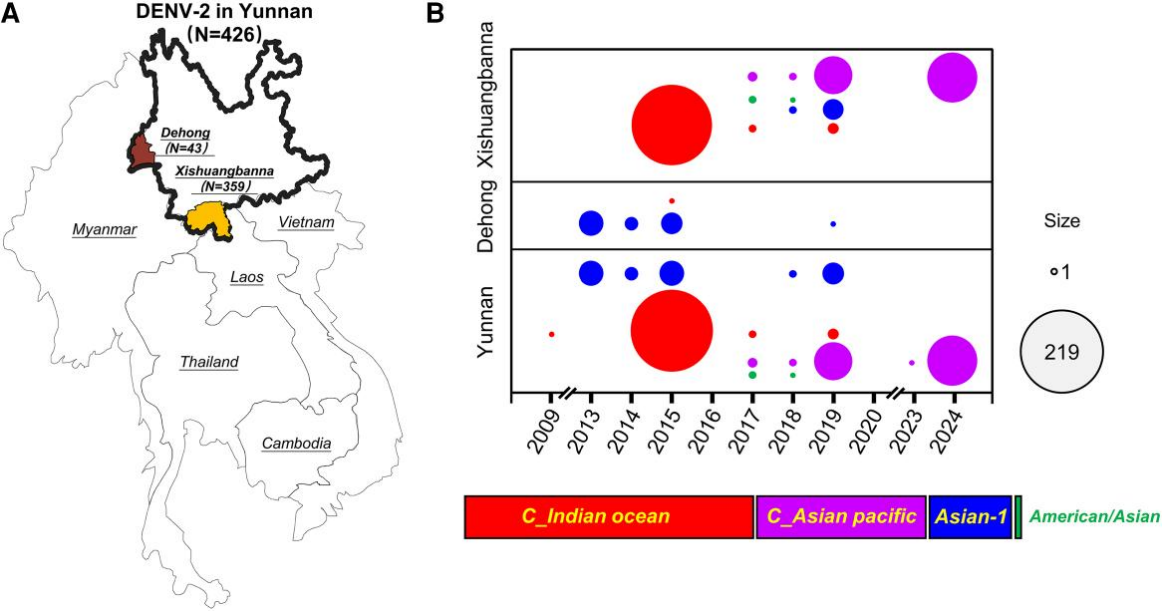
Diversity and distribution characteristics of DENV-2 genotypes in Yunnan, China, 2009–2024. (*A*) The geographical locations of 426 sampling sites for DENV-2 E gene sequences in Yunnan, China. (*B*) The circulating DENV-2 genotypes in 2 regions, Xishuangbanna and Dehong, from 2009 to 2024. Different colored dots represent different genotypes as indicated in the legend, with the size of the dots corresponding to the number of sequences collected.

In summary, we have outlined the diversity and geographic distribution characteristics of DENV-2 genotypes in Yunnan from 2009 to 2024 based on 426 sequences, mainly including 43 from Dehong and 359 from Xishuangbanna (Figure 5*A*). The results indicate that DENV-2 in Yunnan is predominantly represented by cosmopolitan genotypes (subtypes C_Indian Ocean and C_Asian Pacific), followed by Asian-1, with sporadic occurrences of the American/Asian genotype. In addition, the genotypes of DENV-2 in Xishuangbanna are more complex and diverse than those in Dehong (Figure 5*B*).

## DISCUSSION

The growing global significance of dengue fever is due to several factors, including climate change, population growth, human mobility, and urban development [1]. Southeast Asia's tropical and subtropical regions are particularly affected as they are the primary endemic areas for dengue fever [16]. With dengue fever's rapid spread and prevalence, DENV shows genetic diversity and complexity [17, 18]. There are 4 major serotypes of dengue virus: DENV-1, DENV-2, DENV-3, and DENV-4. Each serotype is further subdivided into multiple genotypes. DENV-1 has 5 genotypes I, II, III, IV, and V. DENV-2 is divided into 6 genotypes: Asian I, Asian II, Southeast Asian/American, Cosmopolitan, American, and Sylvatic. DENV-3 consists of 5 genotypes labeled I through V. DENV-4 includes 4 genotypes categorized as I, II, III, and 1 sylvatic genotype [19]. Recent studies have further subdivided the I genotype of DENV-1 into 13 subtypes, the IV genotype into 8 subtypes, and the V genotype into 18 subtypes [20, 21]. The cosmopolitan genotype of DENV-2, which has the largest number of samples, shows considerable complexity in its diversity [22–24]. Depending on the geographical region, the cosmopolitan genotype can be divided into 3 main lineages: Indian Ocean, West African, and Asian Pacific. In this study, we used the latest typing rules to identify dengue virus genotypes and subtypes [22]. Our results showed that the dengue viruses circulating in Xishuangbanna, Yunnan Province, China in 2024 belonged to the C_asian-pacific subtype of DENV-2 and the 1L subtype of DENV-1.

Notably, this study confirmed 3 cases of DENV-1 infection. However, due to the low viral load in the samples, only 1 complete genome sequence of the DENV-1 1L subtype was successfully obtained. Further analysis using BLAST comparison revealed that this sample shares a sequence identity of 99.75%–99.84% with circulating DENV-1 strains (PP563832 and PP563827) from Yunnan, China in 2023. Additionally, the DENV-1 serotype was the predominant strain circulating in the Xishuangbanna region of Yunnan Province in both 2019 and 2023 [10, 25]. This finding suggests that the DENV-1 serotype may have established a relatively stable transmission chain within the “Aedes mosquito-human” vector-host system in this region.

The most significant finding of this study is the identification of 2 new lineages of DENV-2 in the 2024 epidemics in Xishuangbanna, Yunnan Province. This suggests that there may be 2 distinct transmission pathways for the virus. Further analysis of whole-genome sequences, focusing on phylogenetic history and spatiotemporal dynamics, confirmed that lineage-1 likely originated in Thailand, while lineage-2 may have originated in Cambodia. This speculation is supported by data from the World Health Organization, which reported a notable increase in dengue cases in Southeast Asian countries, including Thailand, Malaysia, Cambodia, Vietnam, and Singapore, in 2024 compared with the same period the previous year [26]. Dengue infections in Thailand reached a record 44 387 cases in the first 6 months of 2024 [27]. Similarly, from January 1 to 22 September 2024, Cambodia reported 13 752 dengue cases, resulting in 37 deaths [28]. In addition, the national border of Xishuangbanna extends 966.29 km, and statistics from January to October 2024 show that nearly 900 000 passengers entered and exited through Yunnan's border ports with Cambodia, Laos, Myanmar, Thailand, and Vietnam, representing a year-on-year increase of 116.3% [29]. Following the implementation of a visa waiver between China and Thailand, the number of tourists entering Yunnan surged [30]. Specifically, from March 1 to 8 August 2024, there were 1863 arrivals from Thailand at Xishuangbanna International Airport, a staggering year-on-year increase of 630.59% [31]. Nevertheless, Yunnan's neighboring countries, including Myanmar, Laos, and Vietnam, are regions where dengue fever is also endemic. There is frequent movement between these countries and Yunnan, including by air and rail. This movement is more significant than travel between Thailand and Yunnan. However, the current study did not include an epidemiological investigation of dengue viruses in these neighboring countries, particularly among cross-border populations. Furthermore, the GenBank database lacks full-length genome sequences of DENV-2 from these countries, which may have biased our analysis of the evolution and origin of the current epidemic strain. Therefore, it is necessary to strengthen molecular epidemiological surveillance and monitor the genotypic diversity of DENV in cross-border populations from these neighboring countries in Yunnan. This is critical for effective prevention and control of dengue fever, especially in the context of increased economic activity, frequent trade exchanges, and high population mobility.

DENV strains can exacerbate epidemics by acquiring single-nucleotide variants, resulting in clade replacement and severe outbreaks [32]. A comparative analysis of the complete genome amino acid sequences of 2 novel lineages of dengue strains identified in this study revealed unique nonsynonymous mutations. Specifically, mutations such as M48I and T52A in the C protein, K57R in the PrE protein, V461A in the E protein, and R174K in the NS1 protein were observed. These amino acid mutations may have arisen from the strains adapting to Aedes aegypti mosquitoes and humans; however, further verification is needed to determine whether these mutations facilitate rapid transmission in *Aedes aegypti* or increase pathogenicity in mammals.

This study statistically analyzed the genetic diversity of DENV-2 in Yunnan by examining the DENV-2 sequences obtained during this research alongside the sequences of local cases from the GenBank database. This study's results revealed 3 DENV-2 genotypes in Yunnan: cosmopolitan, Asian-1, and American/Asian. Of these, genotype cosmopolitan was the most prevalent and exhibited significant genetic diversity, including 2 subtypes, C_Indian Ocean and C_Asian Pacific. The Xishuangbanna region in Yunnan exhibited the highest complexity and diversity in DENV-2 genotypes, while the Dehong region displayed more homogeneous genotypes, primarily the Asian-1 type. This observation indicates that the Xishuangbanna region is likely the primary endemic area for DENV-2 in Yunnan. Consequently, it is imperative to enhance molecular epidemiological investigations of DENV-2 in the Xishuangbanna region and implement targeted prevention and control measures. The notable geographical variations in DENV-2 genotypes within Yunnan may be attributable to the predominance of sequences from the Dehong and Xishuangbanna regions in the GenBank database, which could introduce bias into the statistical outcomes. Consequently, a comprehensive epidemiological investigation of DENV-2 genotypic diversity across larger populations in various prefectures of Yunnan Province is necessary. To improve the prediction of dengue fever incidence in Yunnan, China, it is also essential to enhance monitoring across the Indian Ocean basin-wide index. This index reflects the average sea surface temperature anomalies in the tropical Indian Ocean and can help inform the planning and stockpiling of effective measures to address potential outbreaks [33].

In conclusion, we identified 2 new lineages of cosmopolitan dengue virus serotype 2 and characterized their amino acid mutations through an epidemiological survey conducted in Xishuangbanna, Yunnan Province, in 2024. These 2 new lineages originated from different transmission routes, 1 from Thailand and the other from Cambodia. Therefore, it is essential to strengthen the prevention and control measures for cross-border dengue fever in Xishuangbanna, Yunnan Province, and to continue the epidemiological study of dengue fever in the border areas of southwest China.

## Supplementary Material

jiaf422_Supplementary_Data

## Supplementary Data

Supplementary materials are available at *The Journal of Infectious Diseases* online (http://jid.oxfordjournals.org/). Supplementary materials consist of data provided by the author that are published to benefit the reader. The posted materials are not copyedited. The contents of all supplementary data are the sole responsibility of the authors. Questions or messages regarding errors should be addressed to the author.
